# Taking the law into your hands: the impact of attention and nonverbal memory on the gestural misinformation effect

**DOI:** 10.1080/13218719.2025.2526462

**Published:** 2025-08-05

**Authors:** Belanna Kalis, Nicole Dargue, Naomi Sweller

**Affiliations:** a School of Psychological Sciences, Macquarie University, Australia; b Autism Centre of Excellence, School of Education and Professional Studies, Griffith University, Australia

**Keywords:** attention, eyewitness testimony, gesture, misinformation, misleading, nonverbal memory

## Abstract

Eyewitness testimonies, a prominent form of legal system evidence, are often flawed. Gestural misinformation effects occur when witnesses misremember events after seeing gestures conveying false information. Stronger memories may be less vulnerable to suggestion. Therefore, factors that increase memory strength, e.g., nonverbal memory capacity and attention, may mitigate misinformation effects. University students (*N* = 93) were told to pay attention or received no attention instructions before watching a confederate perform a series of actions. Participants were then questioned, with questions accompanied by accurate or misleading gestures, or no gestures, followed by a nonverbal memory test. Correct answers were more likely after viewing accurate gestures than misleading or no gestures. Participants instructed to pay attention gave more correct answers and were misled by gestural misinformation at lower rates than participants not instructed to pay attention. Results suggest gestures can impact eyewitness testimonies, especially if the witness was not paying attention to the crime.

Eyewitness testimonies are an influential form of evidence in the legal system (Howe et al., 2018). Such testimonies are often given more weight than other forms of evidence (Wang et al., 2018), and jurors are more likely to give a guilty verdict if a witness testifies (Loftus, 1975b). However, eyewitnesses are prone to error and are the largest contributing factor to the conviction of innocent people (Gross et al., 2005; Wang et al., 2018; Wells & Olson, 2003).

Eyewitnesses are likely to be flawed because human memory is fallible. The construction hypothesis (Loftus, 1975a) explains that memories are malleable, which means that remembering an event does not merely involve retrieving an exact replica of the event. Rather, memories can be modified. For instance, memories can be altered to incorporate incorrect information that the person is exposed to after the event, which is known as the misinformation effect (Howe et al., 2018; Loftus, 2004; Schacter, 2012).

Numerous studies have demonstrated the misinformation effect. In their seminal study, Loftus and Zanni (1975) had participants watch a video of a car accident and then answer a questionnaire about it. In the questionnaire, the participants were either asked if they saw *a* broken headlight or *the* broken headlight. Those who were asked if they saw *the* broken headlight were more likely to report that there was a broken headlight, even though there was not, which demonstrates that simply implying the presence of an object through wording can distort recall. Similarly, questions that insinuated the presence of a logo, gloves, or a gun prompted participants to erroneously report seeing these features in a video (Sharman & Powell, 2012; Zaragoza & Mitchell, 1996). Moreover, Searcy et al. (2000) had participants watch a video of a murder and then listen to a narrative that described the crime, where some versions of the narrative falsely stated that the culprit had a chipped tooth. Those who heard this misleading detail were more likely to select a suspect with a chipped tooth from a line-up. Likewise, participants were more likely to incorrectly report that a criminal in a video had blue eyes (Zajac & Henderson, 2009) or a tattoo (Eisen et al., 2017) when a confederate misleadingly told them this information.

## The gestural misinformation effect

Although most research into the misinformation effect has focused on verbal misinformation, misinformation can be introduced through other forms, such as images (Braun-Latour et al., 2004; Frenda et al., 2013; Garry et al., 2007; Sacchi et al., 2007; Strange et al., 2008; Wade et al., 2002) and newspaper articles (Otgaar et al., 2009). Research has even suggested that misinformation can be portrayed through gestures. Gestures are movements made by the arms and hands (Levy & McNeill, 1992). However, gestures are more than arbitrary movements. Gestures can convey substantive information, either in addition to or independently of speech (Beattie & Shovelton, 1999; Goldin-Meadow, 2014; Hostetter, 2011; McNeill et al., 1994). Hence, people glean information from gestures and incorporate this information into the communicator’s message (Goldin-Meadow, 1998; Goldin-Meadow & Sandhofer, 1999; Kelly & Church, 1998). For instance, Kelly et al. (1999) found that when the phrase ‘my brother went to the gym’ was accompanied by a basketball gesture, a significant number of participants recalled that the brother was playing basketball, even though this information was only shown gesturally. Therefore, as people extract information from gestures, gestures may provide a source of misinformation if they express misleading details. Accordingly, the gestural misinformation effect occurs when people misremember an event after seeing gestures that convey false information (Gurney et al., 2013).

In the first study of the gestural misinformation effect, Broaders and Goldin-Meadow (2010) had children watch a live musical performance. They then interviewed the children about the performance five times over a 10–12-week period, but some interview questions were accompanied by a gesture that conveyed an incorrect answer. A significant number of children affirmed at least one misleading suggestion made through gestures, and the impact of the misleading gestures on recall carried over to later interviews, illustrating the long-lasting nature of gestural misinformation.

Gurney et al. (2013), Gurney (2015), and Gurney et al. (2016) examined the gestural misinformation effect in adults. In these studies, participants watched a video of a crime and were then questioned about it. Questions were accompanied by a gesture that portrayed the correct answer, a gesture that conveyed incorrect information, or no gesture. Participants were more likely to give the correct response when the question contained an accurate gesture, compared to a misleading gesture or no gesture. Furthermore, participants were more likely to give an incorrect answer that aligned with the misinformation when they saw a misleading gesture, compared to an accurate gesture or no gesture. Gurney (2015) and Gurney et al. (2016) compared gestural misinformation with verbal misinformation by including questions where the misinformation was conveyed through speech, rather than a gesture. Gestural suggestions and verbal suggestions had similar levels of influence, suggesting gestural misinformation may be as powerful as verbal misinformation.

One possible explanation for the gestural misinformation effect is that people presume that the gestures are signalling the correct answer (Gurney et al., 2013). However, Kirk et al. (2015) showed that the gestural misinformation effect is not just due to suggestive pressure. In this study, participants were more likely to incorporate accurate gestures into their recall than misleading gestures, suggesting people do not indiscriminately concur with all presented gestures. The gestural misinformation effect is therefore not just an artefact of demand characteristics, and people do not uncritically comply with interviewers’ suggestions. Furthermore, Gurney et al. (2016) found that most participants did not report feeling misled by gestures, which indicates that they were not intentionally agreeing with the suggestions. As such, it is unlikely the gestural misinformation effect is merely the result of suggestive pressure.

Alternatively, the gestural misinformation effect can be accounted for by source monitoring errors (Johnson et al., 1993). As gestures are a visual source of information, they can emulate the imagery of the witnessed scene, causing people to confuse the details conveyed through gestures with what they actually saw (Gurney et al., 2013). Hence, while seeing accurate gestures can cue an individual’s memory of the event (Kirk et al., 2015), misinformation from inaccurate gestures may be misattributed as being from the original memory.

## Preventing the gestural misinformation effect

Considering that gestural misinformation could impact legal proceedings, it is important to identify safeguards that can reduce the effects of misleading gestures. To examine this idea, Kirk et al. (2015) explored whether factors that protect children against verbal misinformation also reduce the gestural misinformation effect. Factors that usually reduce the verbal misinformation effect in children, such as higher language ability and older age (Bruck & Melnyk, 2004; Clarke-Stewart et al., 2004; Kulkofsky & Klemfuss, 2008), did not prevent the gestural misinformation effect. Hence, it is possible that the gestural misinformation effect may be more resistant to safeguards than the verbal misinformation effect. However, Kirk et al. (2015) only examined factors relevant to children’s suggestibility, and there is a limited understanding of potential safeguards applicable to adults.

The memory trace strength theory (Pezdek & Roe, 1995) explains that weaker memories are more vulnerable to suggestion, because there is more opportunity for false information to be integrated into incomplete memories. Conversely, when the memory trace is strong, individuals are more likely to detect discrepancies between the misinformation and the original event. In this way, factors that increase the strength of the original memory may protect against gestural misinformation.

One such factor could be paying attention to the event. Poor attention disrupts memory encoding, because when people pay less attention, they are unable to process all the details of the scene, leading to impoverished memories (Lane, 2006; Murphy & Greene, 2016; Zaragoza & Lane, 1998). Accordingly, people who pay less attention can perform worse on recall tests (Craik et al., 1996; Murphy & Greene, 2016), meaning that they may be more suggestible. In support of this claim, people who have divided attention (Lane, 2006; Zaragoza & Lane, 1998) or a high attentional load (Murphy & Greene, 2016) during encoding are more susceptible to written misinformation. However, there is currently no research examining whether attention also affects vulnerability to gestural suggestion. Given that prior studies of gestural misinformation were limited because participants were told to pay attention (Gurney, 2015; Gurney et al., 2013, 2016), the present study examined whether being warned to pay attention impacts the gestural misinformation effect.

Another potential safeguard against gestural misinformation is an individual’s memory ability. People who perform better on memory tests, such as tests of facial memory, working memory, and memory for stories, tend to have more accurate recall, as they can retain information more easily (Andersen et al., 2014; Henry et al., 2017). Hence, individuals with higher memory capacities could potentially be less susceptible to misinformation, as they are likely to remember more about the event. In support of this idea, individuals have been found to be less vulnerable to written misinformation if they score higher on memory tests, such as the Operation-Word Span Task, Wechsler Memory Scale, and 2-Back Working Memory Task (Jaschinski & Wentura, 2002; Zhu et al., 2010). It is possible that better memory ability may safeguard against gestural misinformation. One type of memory that may be particularly relevant to gestural misinformation is nonverbal memory, because gestures are a visual source of information. Research has shown that higher nonverbal memory capacity is associated with lower susceptibility to misleading questions for children with intellectual disabilities (Henry & Gudjonsson, 2003). The present study therefore examined whether nonverbal memory capacity affects susceptibility to gestural misinformation for adults.

## The current study

While the source monitoring framework (Johnson et al., 1993) explains that misleading gestures can distort recall, the memory trace strength theory (Pezdek & Roe, 1995) suggests that this effect can be reduced by strengthening the original memory. Prior research indicates that higher memory capacity (Jaschinski & Wentura, 2002; Zhu et al., 2010) and attending to the event (Lane, 2006; Murphy & Greene, 2016; Zaragoza & Lane, 1998) are safeguards against written misinformation. Thus, higher nonverbal memory ability and paying attention may also be protective against gestural misinformation. Hence, the present study aimed to determine whether paying attention and higher nonverbal memory capacity mitigate the gestural misinformation effect.

In the present study, a confederate completed a series of scripted actions. Half of the participants were told they should pay close attention to the confederate, as they would be questioned about the confederate’s actions later. The remaining participants were not given any instruction to pay attention or warning about the confederate. Afterwards, participants were questioned about the confederate, and questions were accompanied by either an accurate gesture, a misleading gesture, or no gesture. Additionally, participants undertook a test of nonverbal memory.

The current study addressed: (a) whether attention and gesture type affect recall accuracy, and whether this is moderated by nonverbal memory ability, and (b) whether attention affects the amount of gestural misinformation that is integrated into recall, and whether this is moderated by nonverbal memory ability. Regarding recall accuracy, it was expected that participants would give more correct answers to questions that had accurate gestures, followed by no gestures, and then misleading gestures, and that participants who were instructed to pay attention would give more correct answers than participants who were not instructed to pay attention. An interaction between gesture type and attention was hypothesised, such that the effect of gesture type on the number of correct responses would be stronger for participants who were not told to pay attention than for those who were told to pay attention. It was further anticipated that nonverbal memory capacity would be positively associated with recall, and an interaction was expected between gesture type and nonverbal memory scores, such that the effect of gesture type on the number of correct responses would become smaller as nonverbal memory scores increased. A further interaction was anticipated between attention and nonverbal memory, such that participants who were instructed to pay attention to the confederate would give more correct responses than participants who were not instructed to pay attention, but that this difference would become smaller as nonverbal memory scores increased. Finally, an interaction between gesture type, attention, and nonverbal memory scores was expected, such that the effect of gesture type on the number of correct responses would be stronger for participants who were not instructed to pay attention than for participants who were instructed to pay attention, but that this difference would become smaller as nonverbal memory scores increased.

Regarding the amount of gestural misinformation that is integrated into recall, it was expected that participants who were instructed to pay attention to the confederate would incorporate fewer misleading gestures into their recall than participants who were not instructed to pay attention, and that participants would incorporate fewer misleading gestures into their recall as nonverbal memory scores increased. Finally, an interaction between nonverbal memory scores and attention was anticipated, such that participants who were instructed to pay attention to the confederate would incorporate fewer misleading gestures into their recall than participants who were not instructed to pay attention, but that this difference would become smaller as nonverbal memory scores increased.

## Pilot study

A pilot study was conducted to determine whether the confederate’s actions, interview questions, and procedure were appropriate for the current study. See Appendix A for pilot study details.

## Method

### Ethics approval and participants

Ethics approval was obtained from Macquarie University’s human research ethics committee (reference code: 520221116136613). A total of 94 undergraduate students from first- and second-year psychology courses at Macquarie University were recruited via an advertisement on Macquarie University’s participant pool website. Participants were offered course credit for participation. To be eligible for this study, participants were required to be fluent in English and to have normal or corrected-to-normal vision. One participant was excluded because they had completed the nonverbal memory test previously, so their results may have been impacted by practice effects. The final sample consisted of 93 participants (71 female, 20 male, one nonbinary, and one unspecified gender), with ages ranging from 17 to 38 (*M* = 20.16, *SD* = 3.93).

Participants were asked their country of birth, with the majority of participants listing Oceania and Antarctica (*n* = 68). For their cultural background, the largest category was Oceanian (*n* = 43). Twenty-three participants were of a non-English-speaking background.

### Experimental design

This study utilised a mixed design with two independent variables and a covariate.

Gesture type was manipulated within subjects and referred to the kind of gesture accompanying each question (accurate gesture, misleading gesture, or no gesture), with five questions accompanied by each gesture type. Attention was manipulated between subjects, with participants either instructed to pay attention to the confederate or not. Allocation to attention conditions was quasi-randomised to ensure an equal number of participants in the attention (*n* = 46) and no attention (*n* = 47) groups. Two measures of nonverbal memory, immediate and delayed nonverbal memory, were numeric predictors. There were two dependent variables: (a) recall accuracy, namely how many correct answers participants gave, and (b) the extent that participants were misled by gestures, namely how many incorrect answers participants gave that corresponded to a misleading gesture.

### Materials

#### Nonverbal memory test

The Rey-Osterrieth Complex Figure Test (ROCFT; Osterrieth, 1944; Rey, 1941) was used to assess nonverbal memory. This test involved three trials: the copy trial, immediate recall trial, and delayed recall trial. In the copy trial, participants replicated a line drawing stimulus. In the immediate recall trial, participants drew the stimulus from memory approximately 3 minutes after the copy trial. In the delayed recall trial, participants drew the stimulus from memory approximately 30 minutes after the copy trial. The copy trial was administered in this study because it is necessary to complete this trial before the other two trials, but it was not used in the final analyses as it is not a measure of nonverbal memory. The ROCFT has adequate internal reliability, with alpha = .80 for immediate recall and .82 for delayed recall (Berry et al., 1991).

#### Distractor task

As the delayed recall trial of the ROCFT must be completed 30 minutes after the copy trial, a distractor task was used as an interlude. The distractor task was a series of riddles (see Appendix B). Riddles were chosen because, following ROCFT instructions, the distractor task must be verbal in nature.

#### Questions

Participants were asked 15 open-ended questions about the confederate (see Appendix C). Each question was designed such that it could be accompanied by either an accurate gesture, a misleading gesture, or no gesture. The type of gesture that accompanied each question was randomised for every participant via the Random Lists online randomiser (https://www.randomlists.com/), so that there were five questions for each gesture type (accurate gesture, misleading gesture, no gesture), but every participant had a different combination of which questions were associated with which gesture type. Question order was randomised for each participant via an online randomiser (https://www.random.org/).

#### Recording and administration

Participant responses were audio- and video-recorded for subsequent scoring and to re-watch the confederate’s performance to verify consistency between participants. Consistency of confederate performance was established by re-watching the videos and noting whether each action performed by the confederate was consistent with the script. The first author watched all videos, and a second rater, blind to participant condition, watched 20% of videos. The confederate performed all actions in the correct order for all participants, with 100% agreement between raters. Participants gave written consent to be recorded. Throughout the study, laptops and a tablet were used to record participants, administer questions, complete the surveys, and time the ROCFT.

### Procedure

The study was conducted in Macquarie University’s School of Psychological Sciences testing spaces. Participants first completed a consent form via Qualtrics (https://www.qualtrics.com), after which the experimenter stated: ‘I just need to grab some materials before we get started. I will be back in a few minutes.’ Those who were in the attention condition were also told ‘while I’m gone, a man will enter the room. You should pay close attention to what he does as you will be questioned about it later.’ Participants in the no attention condition were not given this additional instruction.

The experimenter then left the room. While the experimenter was absent, a confederate entered the room and performed a series of actions, such as sharpening a pencil and stubbing his toe (see Appendix D for the confederate’s actions). The performance lasted approximately 2 minutes. The same confederate was used for all participants, and the confederate was blind to the attention condition allocation of the participants. Once the confederate finished their actions, they left the room and the experimenter re-entered. The participants then completed the copy and immediate recall phases of the ROCFT. Following this, participants were questioned about the confederate’s actions, with each question asked with either an accurate gesture, a misleading gesture, or no gesture. Participants then completed a demographic questionnaire via Qualtrics. After completing the questionnaire, participants were asked riddles until approximately 30 minutes had elapsed since the copy phase of the ROCFT. Participants then completed the delayed recall phase of the ROCFT. The participants were then informally questioned about how they found the experiment and whether they had previously completed the ROCFT. Finally, participants were verbally debriefed about the true purpose of the study, asked to read a debrief statement, and asked to complete a reconsent form via Qualtrics. All participants reconsented. Participants were emailed a copy of the debrief statement, and participants in the second-year psychology course were also sent a summary of the experiment, as per request from the course convenor. The study took approximately 50 minutes per participant.

### Scoring

Participants’ interview answers were coded using the ELAN software Version 6.3 (Max Planck Institute for Psycholinguistics, 2022). For recall accuracy scores, participants were given a point if their answer correctly identified the relevant part of the confederate’s performance. Participants received no point if their answer was incorrect, or no response was given. Scores could range from 0 to 15. For the number of times misled, participants were given a point each time they gave an incorrect response that corresponded to a misleading gesture. No participants gave a response that corresponded to a misleading gesture following either an accurate gesture or no gesture, meaning that participants who were misled were all misled by the gestures themselves, rather than being otherwise mistaken. As each participant received five questions with a misleading gesture, scores could range from 0 to 5.

The ROCFT files were scored according to the standardised protocol (Meyers & Meyers, 1995; Strauss et al., 2006). Participants were scored based on how many elements of the stimulus they replicated correctly. A score out of 36 was given for each of the immediate recall and delayed recall trials, where higher scores indicated better nonverbal memory ability.

### Reliability

To test interrater reliability, a second coder independently scored 20% of the interview answers and ROCFT files using the same scoring method as the original coder. The second coder was blind to participants’ group allocations. The original and reliability coders had complete agreement for the recall accuracy scores and number of times misled scores. For the ROCFT scores, mixed model intraclass correlation coefficients (ICC) were used to calculate absolute agreement between the coders. Interrater reliability was high for both immediate recall scores, *ICC* = .99, *p* < .001, and delayed recall scores, *ICC* = .98, *p* < .001.

## Results

Stata Version 17 was used for all analyses. The immediate and delayed nonverbal memory scores were mean-centred prior to analysis. Preliminary analyses were used to test the assumptions of ANCOVAs. Based on the Shapiro-Wilk test, the assumption of normality was violated for the number of times misled, *W* = .90, *p* < .001. There was a strong positive skew, with very few participants being misled two or more times (see Table 1). As the skew was substantial, bootstrapping would have been inadequate to manage the departure from normality. Analyses addressing the extent to which participants were misled were therefore analysed with a poisson regression. Given the high frequency of zeros in the misled count variable, a negative binomial regression was initially run to test the alpha parameter, to determine whether the data were over-dispersed. The data were found not to be over-dispersed, meaning the poisson model for count data was the appropriate model. The number of times participants were misled was the dependent variable, attention was a categorical predictor, and nonverbal memory was a numeric predictor. This analysis was repeated for immediate and delayed nonverbal memory scores. All other assumptions were met, and the mixed design ANCOVAs for recall accuracy were conducted as planned.

**Table 1.** Number of times participants were misled by gestures according to attention condition.

Misled (*N*)	Attention	No attention
0	33	18
1	12	19
2	1	8
3	0	2
4	0	0
5	0	0

### Main analyses

#### Effect of gesture type, attention, and nonverbal memory on recall accuracy

To examine the first research question, a mixed design analysis of covariance (ANCOVA) with interactions was run using recall accuracy as the dependent variable and gesture type, attention, and nonverbal memory as the predictors. The analysis was run twice using immediate nonverbal memory scores and then delayed nonverbal memory scores. Follow-up pairwise comparisons were Bonferroni adjusted.

First, the analysis was run using immediate nonverbal memory scores (*M* = 21.62, *SD* = 6.00). The main effect of gesture type on recall accuracy was significant, *F*(2, 178) = 24.48, *p* < .001, η_p_^2^ = .22. Pairwise comparisons showed accurate gestures as leading to significantly more correct answers than either no gestures, *t*(178) = 5.90, *p* < .001, η_p_^2^ = .16, or misleading gestures, *t*(178) = 6.21, *p* < .001, η_p_^2^ = .18. However, there was no significant difference between misleading gestures and no gestures, *t*(178) = 0.30, *p* = .76, η_p_^2^ = .00 (see Table 2 for descriptive statistics). There was a significant main effect of attention on recall accuracy, *F*(1,178) = 44.29, *p* < .001, η_p_^2^ = .33, such that participants who were told to pay attention had more accurate recall than participants who were not told to pay attention. The main effect of immediate nonverbal memory scores on recall accuracy was nonsignificant, *F*(1,178) = 0.02, *p* = .90, η_p_^2^ = .00. No interactions were significant, including the interaction between attention and gesture type, *F*(2,178) = 2.30, *p* = .10, η_p_^2^ = .03, the interaction between gesture type and immediate nonverbal memory scores, *F*(2,178) = 1.45, *p* = .24, η_p_^2^ = .02, the interaction between attention and immediate nonverbal memory scores, *F*(1, 178) = 1.56, *p* = .22, η_p_^2^ = .02, and the three-way interaction between gesture type, attention, and immediate nonverbal memory scores, *F*(2,178) = 0.02, *p* = .98, η_p_^2^ = .00.

**Table 2.** Recall accuracy by attention condition and gesture type.

Gesture type	Attention		No attention		Total	
	*M*	*SD*	*M*	*SD*	*M*	*SD*
Accurate	3.56	1.11	2.91	1.04	3.24	1.12
Misleading	2.91	1.31	1.53	1.06	2.22	1.37
None	2.65	1.12	1.89	1.29	2.27	1.26
Total	3.04	1.24	2.11	1.27	2.57	1.34

The ANCOVA examining the effects of gesture type, attention, and nonverbal memory capacity on recall accuracy was then repeated for delayed nonverbal memory scores (*M* = 22.23, *SD* = 5.86). The same pattern of results was found. First, the main effect of gesture type was significant, *F*(2, 178) = 24.72, *p* < .001, η_p_^2^ = .22. Pairwise comparisons showed accurate gestures led to significantly more correct answers than no gestures, *t*(178) = 6.24, *p* < .001, η_p_^2^ = .18, and misleading gestures, *t*(178) = 5.92, *p* < .001, η_p_^2^ = .16. However, there was no significant difference between misleading gestures and no gestures, *t*(178) = 0.32, *p* = .75, η_p_^2^ = .16. The main effect of attention on recall accuracy was significant, *F*(1, 178) = 42.87, *p* < .001, η_p_^2^ = .33, with participants who were told to pay attention giving more correct responses than participants who were not told to pay attention. The main effect of delayed nonverbal memory scores on recall accuracy was nonsignificant, *F*(1, 178) = 1.05, *p* = .31, η_p_^2^ = .01. No interactions were significant, including the interaction between gesture type and attention for recall accuracy, *F*(2,178) = 2.39, *p* = .10, η_p_^2^ = .03, the interaction between gesture type and delayed nonverbal memory scores, *F*(2,178) = 1.89, *p* = .15, η_p_^2^ = .02, the interaction between attention and delayed nonverbal memory scores, *F*(1,178) = 3.61, *p* = .06, η_p_^2^ = .04, and the interaction between gesture type, attention, and delayed nonverbal memory scores, *F*(2, 178) = 0.14, *p* = .87, η_p_^2^ = .00.

#### Effect of attention and nonverbal memory on the likelihood of being misled

For the second research question, to examine whether attention and nonverbal memory capacity affect susceptibility to gestural misinformation, due to the highly skewed nature of the dependent variable, a poisson regression was conducted, with attention and nonverbal memory as predictors. First, the analysis was run with immediate nonverbal memory scores. The main effect of attention was significant, χ^2^(1) = 9.87, *p* = .002, such that the rate of being misled was 2.69 times as high for participants who were not told to pay attention than for participants who were told to pay attention (see Table 1). The main effect of immediate nonverbal memory was nonsignificant, *z =* 1.07, *p = .*29, 95% CI [–0.01, 0.04], as was the interaction between attention and immediate nonverbal memory, χ^2^(1) < 0.01, *p = .*97.

The poisson regression was then repeated for delayed nonverbal memory scores. The same pattern of results was found, with a significant main effect of attention, χ^2^(1) = 11.04, *p* = .001, a nonsignificant main effect of delayed nonverbal memory scores, *z =* 0.41, *p = .*69, 95% CI [–0.01, 0.02], and a nonsignificant interaction between attention and delayed nonverbal memory scores, χ^2^(1) = 0.07, *p = .*80. Again, the rate of being misled was 2.81 times as high for participants who were not instructed to pay attention than for participants who were instructed to pay attention (see Table 1).

## Discussion

The current study investigated whether paying attention and higher nonverbal memory capacity mitigate the gestural misinformation effect. We asked: (a) whether attention and gesture type affect recall accuracy, and whether this is moderated by nonverbal memory ability, and (b) whether attention affects the amount of gestural misinformation that is integrated into recall, and whether this is moderated by nonverbal memory ability. There was an effect of gesture type and attention on recall accuracy, whereby accurate gestures and paying attention both improved recall. Furthermore, participants who were told to pay attention had lower rates of being misled than participants who were not told to pay attention. No effects, however, were moderated by nonverbal memory capacity, and the effect of instructions to pay attention did not moderate the effect of gesture type.

### Recall accuracy

It was expected that participants would give more correct answers to questions that had accurate gestures, followed by no gestures, and then misleading gestures. While participants did show better recall for questions with accurate gestures compared to no gestures and misleading gestures, there was no significant difference between the no gesture and misleading gesture conditions. While the former finding suggests accurate gestures can cue the original memory of the event (Kirk et al., 2015), the latter result suggests that although participants were misled by the misleading gestures (see below), such gestures did not affect overall accuracy of recall. This finding differs perhaps from the expectations of the source monitoring framework (Johnson et al., 1993). It is possible that, in the current study, the misleading gestures did not lead to source confusion. The gestures used by the interviewer in this study were rehearsed, and therefore may not have been as subtle as in real-life conversations. As such, participants may have been consciously aware of the gestures, allowing them to reject the misinformation because source monitoring errors are less likely to occur when the source attribution involves conscious deliberation (Johnson et al., 1993; Lindsay, 1990).

Unexpectedly, there was no relationship between nonverbal memory scores and recall. While individuals who perform better on memory tests have previously been shown to have better recall (Andersen et al., 2014; Henry et al., 2017), prior studies examined facial memory, working memory, and memory for stories. Conversely, the current study tested nonverbal memory. Although the interview questions in this study were based on visual aspects of the confederate’s performance, nonverbal memory may not have been the only type of memory that was relevant to recalling the event. Indeed, the confederate’s performance and the subsequent interview were multimodal, as they involved both verbal and nonverbal components. Future research should incorporate, for example, verbal memory, to gain a more comprehensive understanding of whether memory capacity affects recall.

While the expectation that participants who were instructed to pay attention to the confederate would have more accurate recall than participants who were not instructed to pay attention was supported, there was no interaction between gesture type and attention for recall accuracy, and no significant interactions of nonverbal memory with either other factor. Hence, accurate gestures and instructions to pay attention both aided recall but showed independent effects. Human memory is fallible (Howe et al., 2018; Loftus, 1975a; Schacter, 2012), and the pattern of means in the current study highlights that people who pay attention are likely to still have imperfect recall. Accurate gestures were useful for cueing the correct answer for participants in both of the attention conditions but did not do so differentially across gesture conditions or across individuals with varying nonverbal memory capacity.

### Likelihood of being misled

Participants who were not told to pay attention to the confederate showed higher rates of being deceived by misleading gestures than participants who were told to pay attention, aligning with prior research that found that attention reduces suggestibility (Lane, 2006; Murphy & Greene, 2016; Zaragoza & Lane, 1998). These findings support the memory trace strength theory (Pezdek & Roe, 1995), as individuals who pay attention to an event form stronger memory traces (Lane, 2006; Murphy & Greene, 2016; Zaragoza & Lane, 1998), making them less vulnerable to suggestion.

As with accurate recall, however, there was no effect of nonverbal memory capacity on the likelihood of being deceived by misleading gestures, and no interaction between instructions to pay attention and nonverbal memory. This could be because the sample of university students may have had higher nonverbal memory abilities compared to other populations, masking any effects of nonverbal memory capacity in this study. This lack of a significant effect of nonverbal memory capacity on likelihood of being deceived does, however, match the null effect for recall accuracy, suggesting that participants with higher nonverbal memory scores did not have stronger memories of the event. With no stronger memory of the event, nonverbal memory capacity was not protective against gestural misinformation. The lack of a moderating effect of nonverbal memory on the instructions to pay attention may have implications for the memory trace strength theory (Pezdek & Roe, 1995), perhaps suggesting that attention is protective against gestural misinformation for people of all nonverbal memory abilities. It has been suggested that people need to be attending to an event in order to encode it (Lane, 2006; Murphy & Greene, 2016; Zaragoza & Lane, 1998), so a lack of attention leaves individuals susceptible to gestural misinformation, regardless of their nonverbal memory capacity.

Alternately, however, it is possible that the misleading gestures misled participants without the participants being aware of them. The gestures may not have been obvious to participants, and it was, rather, the subconscious processing of the gestures that resulted in participants being misled by them. If gestures misled participants without them being consciously aware of the gestures, then it is perhaps not surprising that visual memory had no protective effect. Indeed, Gullberg and Kita (2009) have demonstrated that the extent to which we encode information conveyed by gestures can be unrelated to our visual fixations on them. If we can process information from a gesture without fixating on it, then it is perhaps unsurprising that nonverbal memory had no moderating effect on the influence of gestures.

Although anecdotal, there is some evidence to suggest that participants may have been processing the gestures unconsciously, as despite just under half of participants being misled by at least one gesture, only seven participants reported noticing the gestures during the debrief at the end of the study. One participant suspected that the study was about gestural misinformation. Although, as noted above, we can encode information without noticing it, these very low rates of participants reporting noticing the gestures goes some way towards explaining both the low rates of being misled by the misleading gestures, as well as the lack of an effect of nonverbal memory.

### Implications

The present study builds on existing research about gestural misinformation (Broaders & Goldin-Meadow, 2010; Gurney, 2015; Gurney et al., 2013, 2016; Kirk et al., 2015) to suggest conditions under which adults are more or less susceptible to misleading gestures. In particular, paying attention to the event was protective against gestural misinformation, but higher nonverbal memory capacity was not. This finding provides some support for the memory trace strength theory (Pezdek & Roe, 1995), as it suggests that paying attention to an event makes people less vulnerable to gestural misinformation. The current findings similarly support the construction hypothesis (Loftus, 1975a), as almost half of participants were misled, suggesting that their memory of the original event was altered by post-event information. However, the lack of a significant difference for recall accuracy between misleading gestures and no gestures perhaps places doubt on the source monitoring framework’s explanation (Johnson et al., 1993) that misleading gestures should prompt more incorrect responses because people confuse the gestural misinformation with what they actually saw.

As the present study raises concerns about the reliability of eyewitnesses, there are practical implications for legal settings. The findings indicate that gestures can affect recall and that even witnesses with better nonverbal memory capacities are vulnerable to gestural misinformation. Furthermore, this study suggests that witnesses who do not pay detailed attention to the crime are more susceptible to misleading gestures. This is particularly problematic because witnesses generally do not closely attend to the crime, as they often have competing demands, are focused on their own safety, and are not given any warning that the crime will occur (Lane, 2006). Given that eyewitnesses could be susceptible to gestural misinformation, the legal system should implement measures that reduce the possible impact of misleading gestures. For instance, forensic interviews should be videotaped to verify whether gestures are used inappropriately during questioning (Broaders & Goldin-Meadow, 2010; Gurney et al., 2013), and legal personnel should be educated about the importance of refraining from incidentally using misleading gestures.

### Future directions

The current study took place in a laboratory, which, by nature, is not as stressful or demanding as witnessing real-world crimes (Lane, 2006; Zaragoza & Lane, 1998). The current study may therefore have overestimated eyewitnesses’ abilities, as people have less accurate recall in stressful environments (Deffenbacher et al., 2004). Future research could use a paradigm that simulates an actual crime, rather than the relatively trivial actions performed by the confederate in the current study.

Furthermore, there was a short time delay between the confederate’s performance and questioning, which does not replicate real forensic procedures, where witnesses are often not questioned until months after the event (Flin et al., 1992). As memory has been shown to depreciate over time (Flin et al., 1992), the short time delay may again have resulted in overstating participants’ recall abilities and understating their likelihood of being misled by gestures. Later studies should conduct multiple interviews at different time points to better recreate the nature of real forensic interviews.

Finally, there were no consequences or incentives for participants’ answers. This differs from real eyewitness situations, where a testimony has implications for the outcome of a trial (Zaragoza & Lane, 1998). Hence, participants in this study may have put less consideration into their answers than would real eyewitnesses, which could have affected their recall. Future research might consider providing consequences or incentives for participants’ answers.

## Conclusion

The current study examined whether participants’ recall accuracy was affected by different types of gestures, attention, and nonverbal memory ability. Additionally, the study explored whether paying attention and higher nonverbal memory capacity can protect individuals against gestural misinformation. Paying attention to the event and viewing accurate gestures improved recall, and participants who were told to pay attention were less likely to incorporate gestural misinformation into their recall than participants who were not told to pay attention. These findings suggest that, while the gestural misinformation effect is robust against nonverbal memory capacity, paying attention could be a safeguard.

## Appendix A.

### Pilot study details

Six participants (one male and five female), all aged 18 years, were recruited for the pilot study via an advertisement on Macquarie University’s participant pool website. Based on the pilot study, it was apparent that some of the questions were unclear, so they were altered to be less ambiguous. First, ‘where did the man put the marker after he took it off the desk?’ was changed to ‘where did the man put the marker immediately after he took it off the desk?’ to be more specific about the timeframe being queried. Second, ‘where did the man put the name tag?’ was updated to ‘where did the man stick the name tag?’ as some participants were confused by the original wording. Furthermore, ‘what did the man do with the piece of paper after he picked it up?’ was altered to ‘what did the man do with the piece of paper before he put it in the draw?’ to clarify the action being queried. Additionally, ‘what did the man do with the sticky note when he picked it up?’ was changed to ‘what did the man do with the sticky note before he put it in the draw?’ to be more precise about the timeframe of interest. ‘Where did the man hurt himself?’ was adjusted to ‘what part of the man’s body did he hurt?’ because some participants were unsure whether the question was asking about how the man hurt himself or what body part he injured. ‘What was the man doing when he walked into the room?’ was updated to ‘what was the man using when he walked into the room?’ as the original question was not specific enough, so it led to vague answers. Finally, "what did the man do with the ruler?” was altered to ‘what action did the man do with the ruler?’ as some participants did not understand the original question. Data from the pilot study was not included in the main analysis, and no participants from the pilot study participated in the main study.

## Appendix B.

### Distractor Task riddles

**Table:**

Riddle	Answer
The more you take, the more you leave behind. What am I?	Footsteps
What room do ghosts avoid?	The living room
What comes once in a minute, twice in a moment, but never in a thousand years?	The letter “M”
What has many keys, but can’t even open a single door?	A piano
What is more useful when it is broken?	An egg
What runs around the whole yard without moving?	A fence
What is black when you buy it, red when you use it, and grey when you throw it away?	Charcoal
A doctor and a bus driver are both in love with the same woman, an attractive girl named Sarah. The bus driver had to go on a long bus trip that would last a week. Before he left, he gave Sarah seven apples. Why?	An apple a day keeps the doctor away
What can you catch but never throw?	A cold
How do you make the number seven even without addition, subtraction, multiplication, or division?	Drop the “S”

*Note.* Riddles were obtained from the Riddles.com website (https://www.riddles.com/).

## Appendix C.

### Interview questions

**Table:**

Question	Accurate gesture	Misleading gesture
What did the man carry out of the room under his arm?	Book	Laptop
What did the man put in the bag?	Water bottle	Money
What was the last thing that the man did before he left the room?	Hand sanitiser	Knock
What action did the man do with the ruler?	Tap	Bite
What was the first thing that the man moved from the desk into the draw?	Hat	Scissors
What was the man wearing on his hand?	Ring	Watch
What was the man using when he walked into the room?	Earphones	Phone
Where did the man stick the name tag?	Arm	Chest
What did the man do with the sticky note before he put it in the draw?	Scrunch	Writing
What did the man take out of the pencil case?	Sharpener	Eraser
What did the man put in the bin?	Wrapper	Tissue
What was the man wearing on his face?	Mask	Glasses
What part of the man’s body did he hurt?	Foot	Elbow
Where did the man put the marker immediately after he took it off the desk?	Pocket	Ear
What did the man do with the piece of paper before he put it in the draw?	Fold	Tear

## Appendix D.

### Details of the confederate’s actions

The confederate’s scripted actions were as follows:

1. Confederate enters the room and states ‘hi, sorry to interrupt. I’ll just be a few minutes’.
2. Confederate drops a muesli bar wrapper into the bin.
3. Confederate puts the water bottle into the bag on the side of the room.
4. Confederate walks to the desk and sits down on the chair.
5. Confederate puts the hat from the top of the desk into the desk draw.
6. Confederate opens up the pencil case and takes out a sharpener.
7. Confederate picks up the pencil from the desk and sharpens it using the sharpener.
8. Confederate puts the sharpener and pencil into the pencil case.
9. Confederate puts the marker from the desk into his shirt pocket.
10. Confederate takes the sticky note off the desk, scrunches it, and puts it in the desk draw.
11. Confederate takes the piece of paper off the desk, folds it into quarters, and then puts it into the desk draw.
12. Confederate takes out the ruler and name tag from the desk draw.
13. Confederate takes the marker out of his shirt pocket.
14. Confederate taps the ruler on the desk.
15. Confederate writes his name on the name tag, using the marker.
16. Confederate sticks the name tag onto his left shoulder.
17. Confederate puts the marker and ruler into the desk draw.
18. Confederate stands up from the chair and then stubs his toe on the corner of the desk.
19. Confederate sanitises his hands using the sanitiser on the desk.
20. Confederate exits the room.
